# Early Endocardial Morphogenesis Requires Scl/Tal1

**DOI:** 10.1371/journal.pgen.0030140

**Published:** 2007-08-24

**Authors:** Jeroen Bussmann, Jeroen Bakkers, Stefan Schulte-Merker

**Affiliations:** 1 Hubrecht Institute, Utrecht, The Netherlands; 2 Interuniversity Cardiology Institute of the Netherlands, Utrecht, The Netherlands; University of Pennsylvania School of Medicine, United States of America

## Abstract

The primitive heart tube is composed of an outer myocardial and an inner endocardial layer that will give rise to the cardiac valves and septa. Specification and differentiation of these two cell layers are among the earliest events in heart development, but the embryonic origins and genetic regulation of early endocardial development remain largely undefined. We have analyzed early endocardial development in the zebrafish using time-lapse confocal microscopy and show that the endocardium seems to originate from a region in the lateral plate mesoderm that will give rise to hematopoietic cells of the primitive myeloid lineage. Endocardial precursors appear to rapidly migrate to the site of heart tube formation, where they arrive prior to the bilateral myocardial primordia. Analysis of a newly discovered zebrafish Scl/Tal1 mutant showed an additional and previously undescribed role of this transcription factor during the development of the endocardium. In Scl/Tal1 mutant embryos, endocardial precursors are specified, but migration is severely defective and endocardial cells aggregate at the ventricular pole of the heart. We further show that the initial fusion of the bilateral myocardial precursor populations occurs independently of the endocardium and *tal1* function. Our results suggest early separation of the two components of the primitive heart tube and imply Scl/Tal1 as an indispensable component of the molecular hierarchy that controls endocardium morphogenesis.

## Author Summary

In its earliest functional form, the embryonic heart of all vertebrates is a simple linear tube consisting of two cell types. An outer muscular cell layer called the myocardium surrounds an inner vascular cell layer called the endocardium that connects the heart to the vascular system. The integration of both cell types is an important step during heart development, but the formation of the endocardial component of the heart tube is poorly understood. Here, we analyze the formation of the endocardium in the zebrafish embryo and show using time-lapse imaging that it is a highly dynamic structure. In addition, we have identified a zebrafish mutant with a specific defect during endocardial development. This defect is caused by a mutation in T cell acute leukemia 1, a gene that—when misexpressed—causes many cases of childhood leukemias. Here, we show an additional role for this gene during heart development. In mutant embryos, both endocardial and myocardial precursors are specified, but integration of both cell types does not occur properly due to a defective migration of the endocardial precursors. Given the many interactions that occur between the endocardium and the myocardium, our results will provide a more comprehensive understanding of heart development.

## Introduction

The primitive heart tube is the first functional organ in the vertebrate embryo and is composed of a myocardial tube lined by an inner endothelial layer called the endocardium. Significant progress has been made towards elucidating the morphogenetic events and transcriptional control underlying patterning of the myocardium [1]. However, the morphogenetic events and the transcription factors involved in early development of the endocardium remain largely undefined. In fact, the specific embryonic origin of the future endocardial cells and their relationship with the future myocardial cells is still unclear [2].

Results obtained using in vitro differentiation of embryonic stem cells [3] and analysis of different mesodermal or cardiac cell lines [4,5] suggest the development of both cardiac lineages from bipotential progenitors during heart field formation and, indeed, bipotential cells have been identified in the early mouse embryo at a single-cell level [3]. However, lineage tracing experiments in the avian embryo have shown restricted myocardial or endocardial potential of precardiac cells, with both types of precursors intermingled during their migration towards the site of the primitive heart tube [6]. A similar sequestration of endocardial and myocardial cells is observed in the zebrafish where cells at late blastula stages give rise to both endocardial and myocardial cells [7]. These cells also give rise to the head vasculature and an anterior population of hematopoietic cells of the primitive myeloid lineage and become restricted in their potential to form either endocardium or myocardium during early gastrulation stages [8].

After endocardial and myocardial precursors have been specified, complex morphogenetic movements occur that shape the primitive heart tube. Again, most studies have focused on the morphogenesis of the myocardium. One of the early markers of myocardial differentiation is the transcription factor *nkx2.5,* expression of which is detected in the zebrafish in bilateral populations starting at the ten-somite stage [9]. Subsequently, these bilateral myocardial fields merge in the midline at the 18-somite stage, with the first contacts occurring in a relative posterior position [10].The myocardial precursors posterior to the first junction as well as the most anterior portions then come in contact with each other, creating a ring with an central circle devoid of myocardial cells that has been suggested to contain the endocardial precursors [10,11]. Around these stages, expression of cardiac contractile genes, such as such as cardiac myosin light chain *(myl7),* starts [12]. Finally, myocardial cells move to the left, and during a complex and poorly understood process, convert the myocardial disc into the primitive heart tube [10].

The basic helix-loop-helix (bHLH) transcription factor Scl/Tal1, hereafter referred to as Tal1, an oncogene originally identified in childhood leukemias, is expressed in endothelial, endocardial and hematopoietic, but not myocardial cells during early murine development [13]. Tal1 forms a transcriptional complex containing Lmo2 to regulate expression of target genes. Gain of function of Tal1 in combination with Lmo2 leads to an expansion of endothelial and hematopoietic cells at the expense of other nonaxial mesodermal components, including myocardial precursors [14], suggesting an important role for these genes during endocardial/myocardial specification and differentiation. Gene targeting studies in the mouse have revealed that Tal1 is essential for the formation of all blood lineages. Tal1 loss of function also leads to considerably less well-defined defects during endothelial development [15–17]. Knockdown of *tal1* in zebrafish showed a conserved function in generating hematopoietic cells. In addition, *tal1* knockdown embryos display defects during arterial versus venous differentiation [18–20].

Here, we analyze early endocardial morphogenesis in the zebrafish, and show that the endocardium appears to arise from the anterior lateral plate mesoderm, from a region that will give rise to hematopoietic cells of the primitive myeloid lineage. From there, presumptive pre-endocardial cells seem to rapidly migrate posterior and towards the midline, where they are later joined by the bilateral myocardial primordia. The myocardial primordia then fuse over the endocardial layer to form the disc that subsequently gives rise to the primitive heart tube. We show that *tal1* has an additional and previously undescribed role in endocardial development. In *tal1* mutants, migration of endocardial but not myocardial precursors is defective, leading to severe outflow tract stenosis and defects in the formation of the primitive heart tube. Our results suggest a separate origin for the two components of the primitive heart tube and show an indispensable role for Scl/Tal1 during endocardial morphogenesis.

## Materials and Methods

### Zebrafish Lines

The *tal1^t21384^* allele was isolated in a genetic screen for ENU-induced mutations affecting vascular development [21]. The transgenic line *TG(fli1a:gfp)^y1^* [22] was obtained from Brant Weinstein (Bethesda, Maryland). The transgenic line *TG(vegfr4: gfp)^s843^* [23] was obtained from Didier Stainier (San Fransisco, California).

### Meiotic Mapping and Sequencing

The *t21384* mutation was mapped to Chromosome 22 using standard simple sequence length polymorphism mapping. For sequencing of the *tal1* gene, genomic DNA was extracted from 12 wild-type (wt) and 12 mutant *tal1^t21384^* embryos. The three coding exons of the zebrafish *tal1* gene were PCR amplified and sequenced on both strands. A mutation in the third coding exon was confirmed in a panel of 580 single mutant and 53 single sibling embryos using PCR with primers tal1_ex3_fw (5′-CAACATTAATGCACATCTTGG-3′) and tal1-ex3-rev (5′-TCTACCTGGTGGTCTTCCTC-3′) and sequenced using primer tal1_ex3_seq (5′-TGGGCGAACAATCAATTTAG-3′).

### Full-Length Sequence of *kdr, flt1,* and *flt4*

We used a unidirectionally cloned, oligo dT–primed SMART cDNA library constructed from 2- and 3-d-old zebrafish larva using Advantage2 DNA Polymerase Mix (Clontech, http://www.clontech.com/). Primers used for identification of *kdr, flt1,* and *flt4* 3′ and 5′ ends are available upon request.

### Phylogenetic and Synteny Analysis

The MEGA3 package was used for phylogenetic analysis [24]. Amino acid sequences were aligned with ClustalW (the resulting alignment is available upon request) and a phylogenetic tree was constructed using a neighbor-joining algorithm. The resulting tree was tested using 1,000 bootstrap resamplings. Pairwise distances were calculated with the PAM substitution matrix. Identification of vertebrate VEGF receptors and synteny analysis was performed using the Ensembl database (http://www.ensembl.org), release 44, April 2007.

### Whole-Mount In Situ Hybridization and Immunohistochemistry

Single and double in situ hybridizations were performed essentially as described [25], except that labeled probes were purified using NucleoSpin RNA clean-up columns (Machery-Nagel, https://www.macherey-nagel.com/) and embryos were transferred to 15-mm Costar Netwells (Corning, http://www.corning.com/) after hybridization. The substrate used in the second color-reaction was INT ([4-iodophenyl]-3-[4-nitrophenyl]-5-phenyl-tetrazolium chloride) (Roche Applied Science, http://www.roche-applied-science.com/). Previously described probes used were *tal1, gata1, spi1, runx1, hey2, flk1/kdra, ephrin B2a, dab2, nkx2.5, amhc,* and *cmlc2* [9,12,26–34]. Probes for *flt4, kdr,* and *flt1* were transcribed from the 5′ part of the respective cDNAs that code for the extracellular domain of the proteins. Immunohistochemistry using anti-GFP (Torrey Pines Biolabs, http://www.chemokine.com/) and anti-tropomyosin (CH1; Sigma, http://www.sigmaaldrich.com/) antibodies was performed as described [35]. Secondary antibodies were Alexa^555^-anti-rabbit and Alexa^647^-anti-mouse (Invitrogen, http://www.invitrogen.com/).

### mRNA Injections

Full length *tal1* mRNA was transcribed from linearized plasmids using the mMessage-mMachine kit (Ambion, http://www.ambion.com/) as described [18]. mRNA injections were done in a volume of 1 nl at the one-cell stage in Milli-Q (http://www.millipore.com/) water at a concentration of 20 pg/embryo.

### Time-Lapse Analysis

Embryos were mounted in 0.25% agarose in a six-well culture plate with a cover slip on the bottom of the well and imaged with a Leica TCS SP confocal microscope (Leica Microsystems, http://www.leica-microsystems.com/) using a 40× dry objective *(vegfr4:gfp)* or 10× dry objective with 2× digital zoom *(fli1a:gfp)*. Maximal *z*-projections of 40–50 slices at 4 μm per slice were compiled using ImageJ software (http://rsb.info.nih.gov/ij/). Time points were recorded every 5 min (*fli1a:gfp*) or 7.5 min (*vegfr4:gfp*) for 6–8 h. A heated stage was employed to keep the embryos at approximately 28.5 °C.

## Results

### A Truncating Mutation in Zebrafish *tal1*

**Figure 1:**
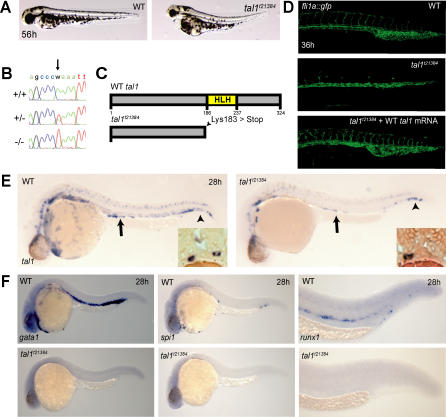
A Truncating Mutation in the Zebrafish *tal1* Gene (A) Live micrographs of wt and *tal1^t21384^* mutant embryos at 56h. Mutant embryos have slightly curved tails, smaller eyes, and pericardial edema. (B) Electropherograms of wt (+/+), heterozygous (+/−) and *tal1^t21384^* homozygous genomic DNA from bp 7–17 of the third *tal1* coding exon. The A→T transversion (arrow) mutates lysine 183 to an ochre (TAA) stop codon (K183X). (C) Schematic diagram of the primary Tal1 protein structure. The DNA-binding bHLH domain is indicated in yellow. The *tal1^t21384^* mutation deletes the Tal1 protein C terminus including the complete helix-loop-helix domain (D) The *tal1^t21384^* mutation leads to severe defects in intersomitic vessel formation at 36 hpf, visualized in a *fli1a:gfp* transgenic background. Early endothelial pattering can be rescued by injecting 20 pg wt *tal1* mRNA into mutant embryos. (E) Loss of *tal1* expression in *tal1^t21384^* mutant embryos. In situ hybridization for *tal1* shows a loss of *tal1* expression in erythroid cells and a reduction of *tal1* expression in the spinal chord. *tal1* expression is retained at normal levels in some hematopoietic/endothelial progenitor cells in the tail (arrowheads) and in some mesenchymal cells of unknown nature just dorsal to the yolk extension (arrows). These bilateral cell populations lie ventral to the pronephric tubules and lateral to the gut endoderm (insets). Also note the loss of *tal1* expression in the ventral wall of the dorsal aorta containing, in wt embryos, the definitive hematopoietic stem cells. (F) Loss of hematopoiesis in *tal1^t21384^* mutant embryos. In situ hybridization for hematopoietic markers at 26 hpf shows a loss of primitive erythroid cells *(gata1),* primitive myeloid cells *(pu.1),* and definitive hematopoietic stem cells *(runx1).*

In a large-scale forward genetic screen [21], we identified a mutant *(t21384)* that showed a severe reduction in endothelial alkaline phosphatase activity at 4 d post fertilization, particularly in the region of the dorsal aorta. Until 26 h post fertilization (hpf), the general morphology of mutant embryos was indistinguishable from that of their wt siblings. At this time however, the pericardium became edematous (Figure 1A), even though heartbeat was initiated normally. No erythrocytes were observed and embryos consequently lacked circulation. To identify the molecular lesion responsible for the *t21384* phenotype, we used simple sequence length polymorphism mapping to position the mutation on Chromosome 22. Single embryo mapping using a limited number of mutant embryos (*n* = 96) positioned the mutation at 28.0 cM (+/− 0.5 cM), between simple sequence length polymorphism markers z21515 and z938, closely linked to the *tal1* gene (27.89 cM)). The combination of endothelial and hematopoietic defects seen in *t21384* mutants had also been observed in mouse Tal1 knockouts [15], as well as in a morpholino knockdown of *tal1* in zebrafish [18–20]. Therefore, *tal1* was a very likely candidate gene. Sequencing of the three coding exons of the zebrafish *tal1* gene in mutant and sibling embryos revealed an A→T transversion in the third and final coding exon (Figure 1B). The mutation resulted in a K→ochre nonsense mutation at position 183 of the protein. The resulting putative protein had a deletion of the highly conserved bHLH domain, including the DNA binding basic region (Figure 1C).

**Figure 2:**
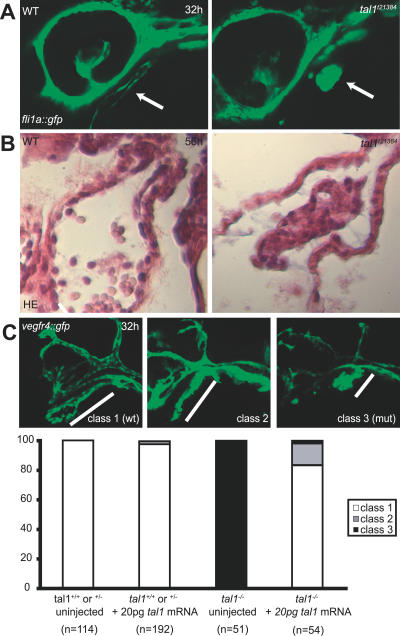
Defects in Endocardial Development in *tal1^t21384^* Mutants (A) Confocal images of the head region from embryos transgenic for *fli1a:gfp* at 32 hpf*.* Arrows indicate the location of the endocardium. In the wt endocardium, a lumen is visible, which is absent in *tal1^t21384^* mutants. (B) Transversal sections of wt and *tal1^t21384^* mutant heart ventricles at 56 hpf stained with hematoxylin/eosin. In wt embryos, the endocardium forms an epithelium attached to the myocardium, and blood cells are visible within the heart tube. In the *tal1^21384^* mutant heart, the endocardium fails to form an epithelium and is only present in the heart ventricle. (C) Rescue of endocardial defects. Maximum *z*-projection of a stack of confocal images of embryos carrying the *vegfr4:gfp* transgene. Embryos were divided into three classes based on their endocardial phenotype. Class 1: wt length of the endocardium with normal tube formation (wt phenotype). Class 2: wt length of the endocardium but no tube formation (intermediate phenotype). Class 3: short endocardium with no tube formed (mutant phenotype). All embryos homozygous or heterozygous for the wt allele are in class 1, whereas all embryos homozygous for the *tal1^t21384^* allele are in class 3, showing the high penetrance of this phenotype. In 43 out of 54 embryos homozygous for the *tal1^t21384^* allele (83%), the endocardial phenotype can be rescued to class 1 through injection of low amounts of wt *tal1* mRNA (20 pg per embryo), whereas most of the remaining (8/54, 15%) display an intermediate phenotype (class 2).

Using this SNP, we also examined the degree of linkage and observed no recombinants for this mutation in 580 mutant embryos tested (genetic distance < 0.09 cM). In genotyping embryos after in situ hybridization or immunohistochemistry, we have never observed a mutant genotype in a sibling embryo. We conclude that the phenotype is fully penetrant (see also Figure 2). To further confirm that the mutation of *tal1* was the defect underlying the *t21384* mutant phenotype, we forced expression of *tal1* by injecting 20 pg of capped wt mRNA into embryos from a cross between heterozygous individuals carrying the *fli1a:gfp* transgene to visualize endothelial cells. In uninjected mutant embryos, vascular patterning was severely affected, leading to a loss of intersomitic vessels. The defect in vascular patterning was rescued by injection of wt *tal1* mRNA (Figure 1D), as was the hematopoietic and the endocardial defect (see below).

Although the *t21384* mutation resided in the final coding exon of the *tal1* gene, and therefore the mRNA was unlikely to be subjected to nonsense-mediated decay [36], we observed reduced expression of *tal1* mRNA at 28 hpf, as revealed by in situ hybridization (Figure 1E). Expression of *tal1* was retained in the ventral mesenchyme of the tail, a region that has been hypothesized to contain hematopoietic progenitors [26]. In addition, we consistently observed a bilateral population of *tal1* expressing cells above the yolk extension. These cells resided in the mesenchyme ventral to the pronephric tubule and lateral to the developing gut tube (Figure 1E, inset).

To confirm the loss of hematopoietic lineages, we performed in situ hybridization for genes that are required for the formation of the two major primitive hematopoietic lineages in the early embryo as well as those that are required for the formation of definitive hematopoietic stem cells. Consistent with data obtained by morpholino injection [18–20], we showed that the development of the primitive erythroid lineage *(gata1)* as well as the formation of definitive hematopoietic stem cells *(runx1)* was lost. Similarly, development of the primitive myeloid lineage was severely affected (Figure 1F), although some *spi1* expressing cells were observed in the head at 28 h (unpublished data).

Based on the tight linkage, the stop codon in the *tal1* gene, and the rescue of all phenotypic aspects in mutant embryos through injection of wt mRNA, we conclude that *t21384* encodes *tal1* and hereafter refer to the mutant allele as *tal1^t21384^*.

### Tal1 Mutants Display Defects in Early Endocardial Development

In addition to the defects observed in the hematopoietic and endothelial lineages (Figure 1D and see below), we observed in *tal1^t21384^* mutants a strong defect in the morphogenesis of the heart that has not been previously described in the zebrafish or mouse. Although myocardial differentiation does not appear to be affected and heartbeat is initiated normally, the endocardial cells in the *tal1^t21384^* mutants do not form a single cell layer lining the myocardium, and do not form atrial endocardium. Instead, endocardial cells aggregate at the arterial pole of the heart, leading to complete ventricular stenosis (Figure 2A and 2B). Later, concentric growth of the myocardium is defective and no heart valves are formed, consistent with an important role for the endocardium in these processes [37,38]. This phenotype was always found in combination with the loss of primitive hematopoiesis, and both aspects of the phenotype could be efficiently rescued by injecting wt *tal1* mRNA, showing the specificity of both phenotypes to the loss of *tal1* function (Figure 2C).

### Early Endocardial Precursors Express a VEGF Receptor Gene That Has Been Lost during Mammalian Evolution

To be able to examine the early endocardial defects observed in *tal1^t21384^* mutants, we aimed to develop several markers for endocardial morphogenesis. A previous study used the expression of a zebrafish vascular endothelial growth factor (VEGF) receptor homologue to delineate early endocardial development in the zebrafish [31], which was proposed to be the zebrafish Flk1/KDR orthologue. However, the murine Flk1 gene is expressed in multiple nonendothelial cells [39,40], and Flk1^+^ progenitors give rise to beating cardiomyocytes [3]. The observed endothelial-specific expression of zebrafish *flk1/kdra* at early developmental stages is therefore surprising. This prompted us to reassess the phylogenetic relationship of the zebrafish VEGF receptors.

**Figure 3:**
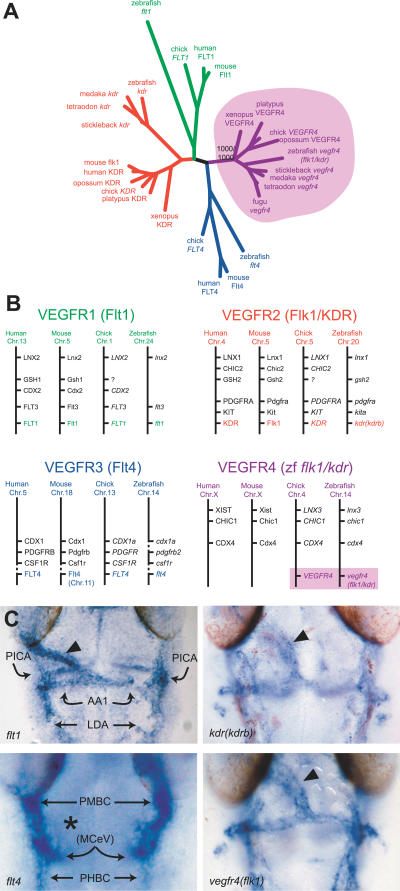
Zebrafish *vegfr4(flk1)* Represents a New Class of Nonmammalian VEGF Receptors (A) Rooted neighbor-joining tree of vertebrate VEGF receptors. Different colors represent different classes of VEGF receptors. Note the clear separation of zebrafish and other teleost fish *vegfr4,* and the novel frog, chick, opossum, and platypus VEGF receptor genes from the three other classes of vertebrate VEGF receptors. Bootstrap value of VEGFR4 node is indicated (1,000 times out of 1,000 iterations). (B) Synteny analysis of vertebrate VEGF receptors using human, mouse, chick, and zebrafish genome assemblies. Colors used are similar to those in (A). Question marks represent missing orthologous genes, potentially due to gaps in the chick genome assembly. Clear syntenic relationships of all vertebrate VEGF receptors were observed, indicating duplication from a primordial gene cluster in a primitive chordate. Mammals have lost the Zebrafish *vegfr4* orthologue, coinciding with the emergence of the X-chromosome inactivation center XIST in the same genomic locus. (C) Expression of zebrafish VEGF receptors in the heart region at 26 hpf, dorsal view, anterior to the top. Three VEGF receptors are expressed in the endocardium: *vegfr4, kdr,* and *flt1* (arrowheads). Note the absence of *flt4* expression in the endocardium (asterisk). At this stage, both *vegfr4* and *kdr* are expressed at high levels in all endothelial cells, whereas *flt1* and *flt4* have a mutually exclusive expression pattern: *flt1* is expressed in all arteries (AA1, first aortic arch; LDA, lateral dorsal aorta; and PICA, primitive internal carotid artery) whereas *flt4* is expressed in all veins (MCeV, middle cerebral vein [which is largely out of the focal plane of this picture]). PHBC, primordial hindbrain channel; PMBC, primordial midbrain channel.

We obtained the full length sequence for all zebrafish VEGF receptors: *flk1/kdra*, *flt1, flt4* and *kdrb* [31,41–43], of which *flk1/kdra* and *kdrb* were proposed to be the result of a whole-genome duplication event in teleost fish [43]. However, we identified likely orthologues for all four zebrafish genes, including both *flk1*/*kdra* and *kdrb* in the genomes of *Xenopus tropicalis,* chick, platypus, and opossum. Phylogenetic and synteny analysis of the three human VEGF receptors FLT1, KDR, and FLT4 and the four receptors of zebrafish, chick, and opossum both supported the hypothesis that zebrafish *flk1/kdra* and the novel chick and opossum VEGF receptor genes in fact represent a separate VEGF receptor class that was lost during mammalian evolution (Figure 3A and 3B). In addition, this showed that the gene previously published as *kdrb* is in fact the KDR orthologue. Given these results, we propose the use of VEGF receptor 4 to designate the novel class of vertebrate VEGF receptors and will use *vegfr4* instead of *flk1/kdra* and *kdr* instead of *kdrb* for the remainder of this manuscript.

Expression analyses revealed that *kdr* was the first VEGF receptor expressed during development (see Figure S1). *vefgr4, flt1,* and *kdr* but not *flt4* are expressed in the endocardium of the heart at 26 hpf (Figure 3C). Importantly, and consistent with earlier results, the expression of *vegfr4* was restricted to endothelial precursors and blood vessels during all stages examined and could be used as a marker for endocardial development.

### Early Endocardial Development in the Zebrafish

To understand the endocardial defects in *tal1^t21384^* mutants, we first characterized normal endocardial development in the zebrafish. We used time-lapse confocal microscopy in the *vegfr4:gfp* transgenic background [23] to analyze the morphogenetic movements during early endocardial development. Consistent with *vegfr4* mRNA expression, this transgene was expressed in the all endothelial cells including the endocardium, but not the myocardium, of the primitive heart tube and therefore was used to follow endocardial precursors during their migration, although it is important to note here that higher resolution tracking of single cells or fate mapping will be required to definitely address the origin and migratory path of endocardial precursors.

**Figure 4:**
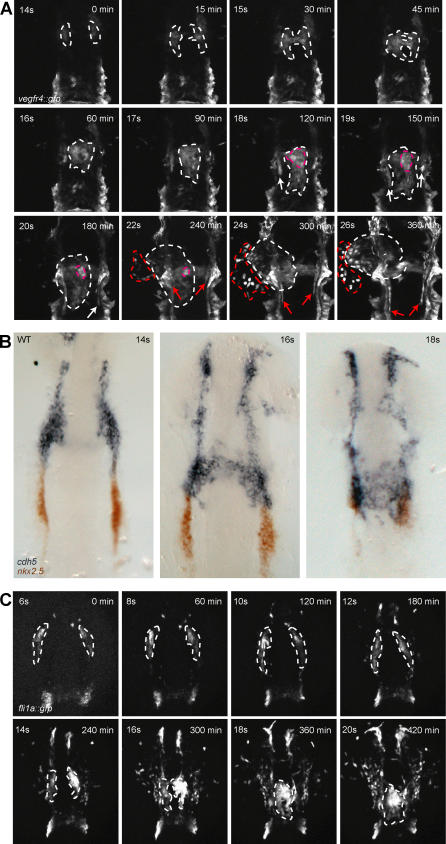
Migration of Endocardial Precursors in wt Embryos (A) Embryos transgenic for *vegfr4:gfp* were subjected to time-lapse confocal microscopy, revealing rapid endocardial migration prior to heart tube formation. A movie demonstrating this process can be viewed in the Video S1. Twelve individual frames from this movie at indicated stages and time-points are shown seen in (A), with white dashed lines indicating the position of (pre-) endocardial cells. Frames 1–5 show the fusion of the bilateral endocardial precursors between the 14- and 16-somite stages. Frames 5–8 indicate the posterior migration of endocardial cells to cover the lateral and posterior regions of the cardiac disc; note the posterior migration of the paired lateral dorsal aortas between the 18- and 22-somite stages (white arrows). The apex of the endocardial disc (pink dashed line) appears to be constricted below the aortic arches between the 18- and 22-somite stages (frames 7–10). A leftward movement of the endocardium is visible between the 20- and 26-somite stages (frames 9–12), and is coinciding with the appearance of single *vegfr4:gfp*-positive cells lateral to the remaining endocardium (red dashed line). Also note the migration of the venous posterior hindbrain channels (red arrows) between the 22- and 26-somite stage (frames 10–12). (B) Relative locations of endocardial and myocardial precursors during fusion of endocardial precursor populations, revealed by two-color in situ hybridization showing *cdh5* (blue, endocardium) and *nkx2.5* (red, myocardium) expression. The bilateral populations of endocardial precursors (arrows) are located anterior to the myocardial precursors until the 14-somite stage, then migrate medially and posteriorly to assume a position in between the myocardial precursors at the 18-somite stage. (C) Embryos transgenic for *fli1a:gfp* were subjected to time-lapse confocal microscopy, revealing slow medial movement of *gfp-*positive cells between the six- and 12-somite stage (frames 1–4) and rapid migration starting at the 14-somite stage. A movie demonstrating this process can be viewed in the Video S2.

The earliest time point at which fluorescence was detected was between the 10- and 12-somite stage (14–15 hpf). At this stage, the transgene was expressed in the anterior and posterior lateral plate mesoderm, representing the endothelial and hematopoietic precursors. Between the 12- and 14-somite stage (15–16 hpf), the anterior lateral plate mesoderm moved medially with more *gfp-*positive cells in the posterior region of the anterior lateral plate mesoderm. This region later formed part of the head vasculature, the primitive myeloid cells, the anterior dorsal aortas and, importantly, the endocardium and aortic arches (see Video S1 and Figure 4A). The coexpression of *vegfr4:gfp* in both the endocardium as well as the head vasculature suggests a respective origin of these cell populations within the anterior lateral plate mesoderm. Both our marker analysis and the time-lapse imaging are consistent with that notion; however, in the absence of single-cell tracking, it is not completely conclusive. Both of these lineages seem to arise as bilateral populations at the 14-somite stage (dashed lines in Figure 4A). The presumed endocardial precursors then rapidly migrated posterior and fused between the 15- and 18-somite stage (16.5–18 hpf). Fusion of these endocardial precursor populations initiated at the anterior side, progressed in a posterior direction, and was finished by 18 hpf. At the same time, further posterior migration of endocardial cells occurred. Finally, a complex leftward movement of the endocardial primordium occurred to position the endocardial component of the primary heart tube at the left side of the embryo between the 22-and 26-somite stage. Although most endocardial cells moved slowly and as an epithelial sheet at these stages, additional single *vegfr4:gfp-*positive cells that are separate from the endocardium were rapidly moving lateral and anterior (Figure 4A, red dashed line).

To assess the relative positions of endocardial and myocardial precursors during fusion of the endocardial precursor populations, we performed two-color in situ hybridization using precisely staged embryos and small time intervals. The endocardial precursors as well as precursors of the head vasculature were marked by expression of *cadherin 5 (cdh5)* and the myocardium by expression of *nkx2.5* (Figure 4B). In this way, we showed that VE-cadherin expressing cells, which include the endocardial precursors, do not express *nkx2.5* and are found immediately anterior to the myocardial precursors in the lateral plate mesoderm at the 14-somite stage, when migration begins. In addition, these data confirmed that endocardial and not myocardial cells are the first to arrive in the midline.

Finally, to confirm the results obtained in the *vegfr4:gfp* transgenic line and to analyze the movements of endocardial precursors before the 14-somite stage, we used the *fli1a:gfp* transgenic line that also expresses a transgene specifically in endothelial cells, including the endocardium (Figure 4C and Video S2). GFP fluorescence in this line can be detected from before the six-somite stage and thus can be used to follow tissue movements prior to endocardial migration. In addition to the endocardium, this transgene is expressed in the developing primitive myeloid population, as well as the pharyngeal mesoderm. Time-lapse imaging of this line confirmed the results obtained with the *vegfr4:gfp* transgenic line between the 14- and 20-somite stage. Before this stage, the anterior lateral plate mesoderm gradually moved medially. This analysis also suggests a close association of endocardial and primitive myeloid cell populations, as macrophages (identified based on their motility and being *fli1a:gpf* positive) start migrating from the 12-somite stage from a region that also includes the endocardial precursors.

### Early Endocardial Development in the *tal1^t21384^* Mutant

**Figure 5:**
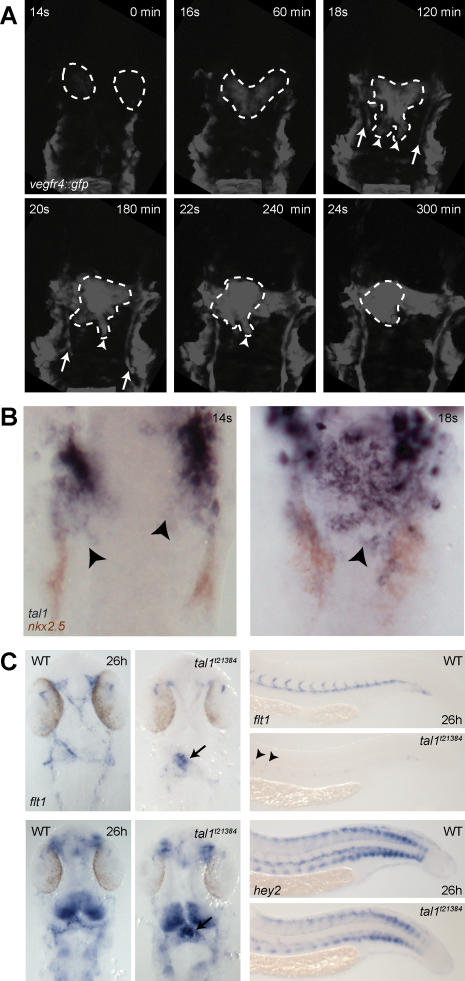
Migration of Endocardial Precursors and Heart Tube Formation in wt *tal1^t21384^* Mutant Embryos (A) *tal1^21384^* mutant embryos transgenic for *vegfr4:gfp* were subjected to time-lapse confocal microscopy, revealing defects during early endocardial precursor migration. A movie demonstrating this process can be viewed in the Video S3. Six individual frames from this movie are shown in (A). Whereas the initial formation of bilateral endocardial precursors is not affected, posterior migration is disturbed, and endocardial precursors remain attached in a relative anterior position. Note that migration of the paired lateral dorsal aortae (arrows) proceeds normally. (B) *tal1* expression in endocardial precursors. Two-color in situ hybridization revealing *tal1* (blue) and *nkx2.*5 (red) expression in wt embryos at the 14- and 18-somite stage. *tal1* expression is observed in endocardial but not myocardial precursors during their posterior migration (arrowheads). (C) Expression of the arterial markers *flt1* and *hey2* is retained in *tal1^t21384^* mutant endocardium, but severely reduced in endothelium, as revealed by in situ hybridization. Dorsal view of *flt1* and *hey2* expression in the head, anterior to the top, and lateral view of *flt1* and *hey2* in the tail (28 hpf). In wt embryos, *flt1* expression is observed in all head arteries, the aortic arches and the endocardium (arrow). In *tal1^t21384^* mutant embryos, expression of *flt1* is observed in a few remaining head arteries and the aortic arches. High levels are seen in the endocardium (arrow). In wt embryos, *flt1* expression is observed in the dorsal aorta and the developing intersegmental vessels. In the tail of *tal1^t21384^* mutant embryos, expression of *flt1* is abolished, except for a few remaining cells that express *flt1* at low levels (arrowheads). In wt embryos, *hey2* expression is observed in the endocardium and the aortic arches and in some parts of the brain and spinal chord. In *tal1^t21384^* mutant embryos, expression in the endocardium (arrow) is increased. In wt embryos, *hey2* expression is observed in the dorsal aorta and the developing intersegmental vessels, spinal chord neurons, and in ventral and dorsal cells of the somites. In *tal1^t21384^*mutant embryos, expression in the dorsal aorta and intersegmental vessels is severely reduced, although some anterior intersegmental vessels and aortic cells retain low levels of *hey2* expression (arrowheads).

To assess the timing of endocardial defects in *tal1^t21384^* mutants, we analyzed mutant embryos carrying the *vegfr4:gfp* transgene and performed time-lapse confocal microscopy, similar to wt embryos (Figure 5A and Video S3). In mutant embryos, fluorescence was first detected between the ten- and 12-somite stage, indistinguishable from wt embryos. The bilateral presumptive endocardial precursors initiated migration at the 14-somite stage normally, but there was a severe defect in the continued posterior migration of endocardial precursors. Whereas wt endocardial precursors migrated rapidly and formed a single cell layer in the embryonic midline, endocardial precursors in *tal1^t21384^* mutants aggregated in an anterior position (Figure 5A and Video S3).

### Tal1 Expression in Zebrafish Endocardial Precursors

Previous reports have indicated Tal1 expression in the endocardium of the mouse [13]. However, a previous study did not identify *tal1* expression in endocardial cells in the zebrafish [26]. We analyzed *tal1* expression during early endocardial development using two-color in situ hybridization and showed *tal1* expression during all stages of endocardial cell migration (10–20-somite stage) (Figure 5B), consistent with a cell-autonomous role for *tal1* in this process. In the trunk, *tal1* expression was detected in angioblasts and primitive erythrocytes. Expression is downregulated during endothelial differentiation and only maintained in erythrocytes. Similarly, expression of *tal1* in the endocardium was downregulated during early migration and maintained in the primitive myeloid lineage.

### Endocardial Differentiation in *tal1^t21384^* Mutants

Results obtained using morpholino knockdown have shown an important role for Tal1 in the differentiation of arterial and venous endothelial cells [18–20]. This suggested that failure of endocardial differentiation could be the primary defect in *tal1^t21384^* mutant hearts. Using the genetic mutant, we reassessed the role of *tal1* during arteriovenous differentiation and showed that indeed most arterial gene expression was lost and venous gene expression expanded (Figures 5C and S2). In addition, we resolved a difference between previous data [18,19] and showed migration of angioblasts to the region of the dorsal aorta not to be affected. Endothelial cells were present in their correct location ventral to the notochord (Figure S3).

Many arterial markers, such as *flt1*, *hey2,* and *dll4* but not venous markers such as *flt4* and *dab2* are also expressed in the endocardium at 24–28 hpf, suggesting common regulation of gene expression. Importantly, we observed that *tal1* differentially regulates arterial and endocardial gene differentiation as expression of *flt1* and *hey2* is severely reduced in the head and trunk arteries, but expression is maintained in the endocardium (Figure 5C). In the absence of more specific endocardial marker genes, these results suggest that defects during migration rather than during endocardial differentiation are the cause of the heart defects observed in *tal1^t21384^* mutant embryos.

### Assembly of Endocardium and Myocardium during Early Heart Tube Formation

**Figure 6:**
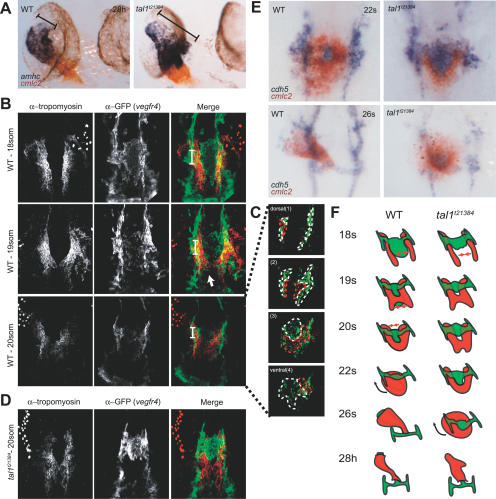
Defective Heart Tube Formation in *tal1^t21384^* Mutant Embryos Despite Normal Fusion of Bilateral Myocardial Precursor Populations (A) Chamber differentiation in wt and *tal1^t21384^* mutant embryos revealed by two-color in situ hybridization showing *amhc* (atrium) and *cmlc2* (atrium and ventricle) at 28 hpf. Chamber differentiation proceeds normal in mutant embryos, but the primary heart tube is malformed with an enlarged atrial inflow region (brackets). (B–D). Two-color immunohistochemistry using anti-tropomyosin (red, myocardium) and anti-gfp (green, *vegfr4,* endocardium) antibodies. Images were generated as maximum projections of confocal *z*-stacks (ventral views, anterior to the top). Some yolk platelets show intense autofluorescence at the wavelength used for anti-tropomyosin detection (647 nm). (B) Heart morphogenesis during heart field fusion in wt embryos. The bilateral heart fields fuse dorsal to the endocardium between the 18- and 20-somite stage. Fusion is initiated in the posterior region of the heart. At the 18-somite stage, tropomyosin-positive cells are located ventral to the first aortic arches. (C) Endocardial precursors are ventral to the myocardium in the lateral and posterior regions of the heart. Endocardial and myocardial sheets are closely associated, as relative positions were only revealed after deconvolution of confocal stacks. Four deconvolved images of the confocal image stack in (B) are shown in (C). (D) In *tal1^t21384^* mutant embryos, initial fusion of the myocardial precursor populations occurs normally, although endocardial precursors are absent in the posterior region of the heart field. (E) Primary heart tube formation from myocardial and endocardial precursors in wt and *tal1^t21384^* mutant embryos revealed by two-color in situ hybridization showing *cdh5* (endocardium, blue) and *cmlc2* (myocardium, red) expression. Dorsal views, anterior is to the top. At the 22-somite stage, wt embryos have formed a cardiac disc, with endocardial cells underlying the circular myocardial primordium. The medial endocardium within the ring of myocardium forms the connection between the endocardium and the aortic arches. In *tal1^t21384^* mutant embryos, anterior closure of the myocardial primordium is defective due to aggregation of endocardial precursors. At the 26-somite stage, wt embryos have formed the primary heart tube and rhythmical contractions begin. In *tal1^t21384^* mutant embryos, heart tube formation is delayed. By this stage, myocardial cells have completed fusion formation at the anterior side of the cardiac disc. (F) Schematic overview of fusion of myocardial precursor regions and heart tube formation in wt and *tal1^t21384^* mutant embryos. Endocardium and aortic arches are in green, myocardium in red.

Heartbeat initiated normally in *tal1^t21384^* mutant embryos, indicating normal myocardial differentiation in the absence of an endocardial lining. Indeed, differentiation of atrial and ventricular myocardium was observed at 28 hpf (Figure 6A). However, at this stage, the atrial side of the heart appeared abnormally enlarged (bracket in Figure 6A), suggesting early defects during heart tube morphogenesis. Therefore, we performed two-color immunohistochemistry labeling the myocardium *(tropomyosin)* and endocardium *(vegfr4:gfp)* in single embryos. Using this method, we confirmed the observation that fusion of the myocardial primordia is initiated in a relative posterior position to form a butterfly-like shape that changes to a horseshoe-shaped myocardium through fusion of the most posterior part of the myocardial primordium between the 18- and 20-somite stages (Figure 6B) [10]. At the onset of myocardial fusion, most of the endocardium is positioned medially to the bilateral myocardial precursors. Connection between the endocardium and the lateral dorsal aortae is persistent throughout development and occurs through the developing first aortic arches. These cells represent the endocardial cells located dorsal to the myocardium as described by Trinh et al. [44] (brackets in Figure 6B). Fusion of the myocardium occurs dorsal to the endocardium and is initiated just anterior to the most posterior endocardial cells (arrow in Figure 6B). Indeed, around this stage, movement of endocardial cells occurs ventrally relative to the myocardial cells, and by the 20-somite stage, most endocardial cells are located ventral to the myocardium—especially in the lateral and posterior regions of the heart (Figure 6C). However, the region of the endocardium that connects to the aortic arches is still positioned medially to the myocardium.

In *tal1^t21384^* mutant embryos, fusion of the bilateral myocardial precursors is initiated normally, and thus appears independent of the endocardium or *tal1* function. Endocardial cells at the 22-somite stage remain located anterior to the myocardium, leading to defects in the anterior fusion of the myocardium, which in wt embryos has occurred at this stage (Figure 6D and 6E). Anterior fusion of the myocardium is delayed until the 26-somite stage (Figure 6E). The medial region of the myocardium at the 22-somite stage gives rise to the ventricle of the heart, whereas the lateral and posterior myocardium gives rise to the atrium [12]. Therefore, absence of atrial endocardial cells can be explained by an early migration defect of endocardial precursors. A summary of heart tube assembly from endocardial and myocardial precursors in wt and *tal1^t21384^* mutant embryos is provided in Figure 6F.

## Discussion

### A Second Genetic Vertebrate Model for Loss of *tal1* Function

We have identified a truncating mutation in the zebrafish *tal1* gene, making this the second species for which a genetic mutation for this important hematopoietic transcription factor is available. The *tal1^t21384^* allele contains a truncating mutation that deletes the conserved bHLH domain of the protein. Functional interaction of Tal1 and Lmo2 is required for the early stages of vascular and hematopoietic lineages in the zebrafish [45], and this interaction occurs at the second helix, a region that is deleted by the *tal1^t21384^* mutation. Moreover, expression of only the helix-loop-helix domain was sufficient to rescue hematopoietic and endothelial development in *tal1* morphants [45], indicating that the N-terminal part of the Tal1 protein is dispensable for early hematopoietic and endothelial development. Therefore, we conclude that the *tal1^t21384^* mutation leads to a complete loss of *tal1* function during early cardiovascular and hematopoietic development.

### Loss of a Fourth VEGF Receptor Gene during Mammalian Evolution

We used a transgenic line under the control of the zebrafish *vegfr4* promoter to follow endocardial development. Surprisingly, *vegfr4* represents a fourth VEGF receptor class with orthologues in all vertebrates—except placental mammals—for which sufficient genome information is available. This fourth VEGF receptor class most likely arose as a consequence of the two proposed whole-genome duplication events that occurred before vertebrate divergence [46]. This is evidenced by the phylogenetic tree of the vertebrate VEGF receptors, which places the emergence of this fourth class prior to the divergence of teleost fish and terrestrial vertebrates. Linkage of class III receptor tyrosine kinases (which includes the VEGF receptors) to a caudal-type homeobox gene is conserved in the basal chordate amphioxus [47], suggesting that this configuration represents the ancestral state.

Our data strongly suggest the loss of a fourth VEGF receptor within the mammalian lineage, as we identified an orthologue of *vegfr4* in the genome of the opossum *Monodelphis domestica,* tightly linked to the Cdx4 gene. This implies that the loss of a mammalian *vegfr4* orthologue occurred relatively recently—after the divergence of the placental (eutherian) and marsupial mammals ∼180 million years ago, but before the mammalian radiation. Interestingly, the human and mouse Cdx4 genes are found adjacent to the XIST noncoding RNA that regulates X-chromosome inactivation. XIST originated through pseudogenization of the LNX3 gene [48], of which an orthologue is also found in the zebrafish *vegfr4-cdx4* locus. The differences between marsupial and placental mammals in the mechanism of X-chromosome inactivation [49], together with the observation that Cdx4 has a role during placental development [50], and the absence of a fourth VEGF receptor in placental mammals present in other vertebrate species (this study) suggest that the *cdx4*–*vegfr4* locus has been an important hotspot during mammalian evolution.

The observation that noneutherian vertebrates have a fourth VEGF receptor has consequences for understanding of the relationship of *tal1* and the VEGF receptors. The murine KDR orthologue, Flk1, was found to function upstream of Tal1, and Tal1 expression was not detected in Flk1^−/−^ embryos [51]. However, the putative zebrafish *flk1* orthologue was found to be downstream of *tal1* during zebrafish hematopoietic development [26], leading to controversy in the understanding of their interactions. Here, we redefine the orthology among the different VEGF receptor classes, and importantly, we identify the true Flk1/KDR orthologue in the zebrafish (*kdr*). We find that early nonendothelial *kdr* expression, which starts 3–4 h before the onset of *tal1* expression, is not affected in *tal1^t21384^* mutants. Therefore, we conclude that *tal1* does not function as an essential factor for the initiation of *kdr* expression. Rather, maintenance of high-level endothelial expression of this gene is dependent on *tal1*.

### Morphogenesis and Embryonic Origins of the Endocardium

Lough and Sugi [2] reviewed data on endocardial morphogenesis in higher vertebrates and proposed that endocardial and myocardial precursors are both derived from the same anatomic location: the cardiogenic mesoderm. From there, endocardial precursors migrate as single cells in between the myocardium and the underlying endoderm. Subsequently, endocardial precursors assemble in two bilateral vascular chords that later on fuse to form the inner lining of the primitive heart tube. Our data show that endocardial morphogenesis in the zebrafish embryo differs in at least two important aspects from that found in higher vertebrates. First, endocardial precursors appear to be derived from a distinct anterior region of the cardiogenic mesoderm, and require posterior migration to the site of heart tube formation. This difference is potentially due to the lack of dynamic observations of endocardial precursor migration in higher vertebrates. Indeed, some data indicate that in higher vertebrates, the endocardium arises from a specific region of the cardiogenic mesoderm [52]. Our results give a first look at the origin of endocardial cells during early development. However, to obtain conclusive evidence regarding the cell movements and origins of the endocardium, higher-resolution fate mapping and cell tracing will be required. Second, zebrafish endocardial precursors do not assemble into bilateral vascular chords, but form a sheet medial to the myocardial precursors. The bilayered disc that is formed through fusion of the myocardial primordia over the endocardium is then converted into the primitive heart tube. This last difference is potentially due to differences in developmental timing between fusion of bilateral primordia and heart tube morphogenesis in zebrafish and higher vertebrates.

Using the *vegfr4:gfp* and *fli1a:gfp* lines and two-color in situ hybridization, we suggest that the endocardium of the primitive heart tube forms from two bilateral precursor populations that are located immediately anterior to the bilateral myocardial precursor populations that express *nkx2.5*. The finding that the endocardium might arise from a region anterior to the myocardial primordia is surprising, given the lineage-tracing experiments performed by Serbedzija and coworkers [53]. In their study, cells were labeled anterior to the *nkx2.5*-expressing myocardial primordia at the ten- to 12-somite stage. At 33 hpf, these cells were found to contribute to the “head mesenchyme” and no cells were found in the heart. One explanation for this finding is that only cells were labeled immediately anterior to the *nkx2.5*-expressing region. Our time-lapse imaging indicates that these cells give rise to the aortic arches and the head vasculature, whereas endocardial precursors originate from a position within the part of the anterior lateral plate mesoderm that is slightly more anterior and ventral. Therefore, Serbedzija and coworkers most likely have not labeled the cells that we show here constitute the endocardial precursors.

Recently, Kattman et al*.* [3] used the differentiation of murine embryonic stem cells to show the existence of a cell population expressing Flk1, with the potential to form both myocardial and endocardial cells in vitro. Our data indicate that in the zebrafish embryo, there are anatomically separate populations of endocardial and myocardial precursor cells during early developmental stages. We show that during normal development both lineages undergo different morphogenetic movements that are suggestive of early lineage separation of endocardium and myocardium. However, this does not imply that these precursors are restricted to one particular differentiation fate at this stage, and given alternative (ex vivo) cues they can still contribute to both lineages. Our data also indicate that anatomically, the endocardial precursors are found in close association with a particular subset of the hematopoietic lineage in the zebrafish: the anterior population of primitive myeloid cells. Lineage tracing experiments will identify whether there exists a lineage relationship with a common endocardial–myeloid progenitor, or whether endocardial and myeloid precursors are simply intermingled during one stage of their development. While resolving this specific question is beyond the scope of this paper, it is worth noting that we observed migrating cells with macrophage morphology that have low levels of *vegfr4:gfp* expression originating from the same bilateral populations of cells as those giving rise to the endocardium and the head vasculature (Figure 4A). This transgene is not expressed in differentiated primitive myeloid cells, and so the signal potentially represents residual *gfp* protein from a previous stage of their development.

### Conclusion

How the endocardial lining of the primitive heart tube becomes established is one of the least-understood aspects of cardiac morphogenesis [2]. In recent years, studies on zebrafish embryos have provided significant insight into the genetic regulation of myocardial development. We show here that a similar approach can be taken to study endocardial development. Given the many interactions between the myocardium and endocardium, both during development as well as in adult physiology and disease [54], our findings will provide a more comprehensive understanding of the morphogenesis and genetic regulation of the heart.

## Supporting Information

Figure S1 *kdr* Expression during Gastrulation At the tailbud (TB) stage, *kdr* but not *vegfr4* is expressed. Expression of *kdr* is diffuse, but highest around the tailbud and near the embryonic axis.
(4.3 MB EPS)

Figure S2 Endothelial Gene Expression in the Trunk Trunk expression of different markers in wt and *tal1^t21384^* mutant embryos at 24–30 hpf for general endothelial *(cdh5, vegfr4),* venous *(flt4, dab2),* and arterial *(notch3, ephrinB2)* differentiation. In mutant embryos, expression of arterial marker genes is reduced or absent, whereas the expression domain of venous marker genes is expanded to the dorsal aorta.
(8.1 MB EPS)

Figure S3 Presence of Endothelial Cells in the Region of the Dorsal Aorta Immunohistochemistry using anti-GFP antibody in the *fli1a:gfp* background; brown labeling indicates *gfp*-positive endothelial cells. Although no luminized vessels in the trunk are formed in *tal1^t21384^* mutants, endothelial cells are present in the location of the dorsal aorta (arrows), and some intersegmental vessels form (arrowhead).
(7.2 MB EPS)

Video S1 Time-Lapse Movie of Normal Endocardial Development in the *vegfr4:gfp* Transgenic Background (4.9 MB MOV)

Video S2 Time-Lapse Movie of Normal Endocardial Development in the *fli1a:gfp* Transgenic Background (2.8 MB MOV)

Video S3 Time-Lapse Movie of Endocardial Development in *tal1^t21384^* Mutants in the *vegfr4:gfp* Transgenic Background (2.2 MB MOV)

### Accession Numbers

The National Center for Biotechnology Information (NCBI) GenBank (http://www.ncbi.nlm.nih.gov/sites/entrez?db=Nucleotide) accession numbers for the nucleotide sequences of the zebrafish VEGF receptors are *kdr,* AY523999; *flt1,* AY524000; and *flt4,* AY5234001.

The NCBI GenBank and Ensembl (http://www.ensembl.org/) accession numbers for the genes and gene products (other than the zebrafish VEGF receptors noted above) used for phylogeny reconstruction are Flk1 (mouse/Mus musculus), NP_034742; *FLT1* (chick/Gallus gallus), NP_989583; FLT1 (human/Homo sapiens), NP_002010; Flt1 (mouse), NP_034358; *FLT4* (chick), XP_414600; FLT4 (human), NP_002011; Flt4 (mouse), NP_032055; KDR (frog/Xenopus tropicalis), ENSXETG00000021061; KDR (human), NP_002244; *kdr* (medaka/Oryzias latipes), GENSCAN00000045289; KDR (opossum/Monodelphis domestica), encoded by ENSMODG00000020673; KDR (platypus/Ornithorhynchus anatinus), ENSOANG00000003802; *kdr* (stickleback*/Gasterosteus aculeatus*), ENSGACG00000014277; *kdr* (tetraodon*/Tetraodon nigrividis*), GSTENG00032761001; *KDR* (chick), NP_001004368; *VEGFR4* (chick), XP_420292; VEGFR4 (frog), GENSCAN00000039479; *vegfr4* (fugu/Takifugu rubripes), SINFRUG00000131563; *vegfr4* (medaka), ENSORLG00000001940; VEGFR4 (opossum), ENSMODG00000020842; VEGFR4 (platypus), ENSOANG00000002222; *vegfr4* (stickleback), ENSGACG00000017117; *vegfr4* (tetraodon), GSTENG00031225001; and *vegfr4* (zebrafish), NP_571547.

## Abbreviations

bHLH: basic helix-loop-helix
hpf: hours post fertilization
wt: wild type

## References

[pgen-0030140-b001] Olson EN (2006). Gene regulatory networks in the evolution and development of the heart. Science.

[pgen-0030140-b002] Lough J, Sugi Y (2000). Endoderm and heart development. Dev Dyn.

[pgen-0030140-b003] Kattman SJ, Huber TL, Keller GM (2006). Multipotent flk-1(+) cardiovascular progenitor cells give rise to the cardiomyocyte, endothelial, and vascular smooth muscle lineages. Dev Cell.

[pgen-0030140-b004] Schultheiss TM, Burch JB, Lassar AB (1997). A role for bone morphogenetic proteins in the induction of cardiac myogenesis. Genes Dev.

[pgen-0030140-b005] Nemer G, Nemer M (2002). Cooperative interaction between GATA5 and NF-ATc regulates endothelial-endocardial differentiation of cardiogenic cells. Development.

[pgen-0030140-b006] Cohen-Gould L, Mikawa T (1996). The fate diversity of mesodermal cells within the heart field during chicken early embryogenesis. Dev Biol.

[pgen-0030140-b007] Lee RK, Stainier DY, Weinstein BM, Fishman MC (1994). Cardiovascular development in the zebrafish. II. Endocardial progenitors are sequestered within the heart field. Development.

[pgen-0030140-b008] Keegan BR, Meyer D, Yelon D (2004). Organization of cardiac chamber progenitors in the zebrafish blastula. Development.

[pgen-0030140-b009] Chen JN, Fishman MC (1996). Zebrafish tinman homolog demarcates the heart field and initiates myocardial differentiation. Development.

[pgen-0030140-b010] Glickman NS, Yelon D (2002). Cardiac development in zebrafish: Coordination of form and function. Semin Cell Dev Biol.

[pgen-0030140-b011] Stainier DY, Lee RK, Fishman MC (1993). Cardiovascular development in the zebrafish. I. Myocardial fate map and heart tube formation. Development.

[pgen-0030140-b012] Yelon D, Horne SA, Stainier DY (1999). Restricted expression of cardiac myosin genes reveals regulated aspects of heart tube assembly in zebrafish. Dev Biol.

[pgen-0030140-b013] Drake CJ, Fleming PA (2000). Vasculogenesis in the day 6.5 to 9.5 mouse embryo. Blood.

[pgen-0030140-b014] Gering M, Yamada Y, Rabbitts TH, Patient RK (2003). Lmo2 and Scl/Tal1 convert non-axial mesoderm into haemangioblasts which differentiate into endothelial cells in the absence of Gata1. Development.

[pgen-0030140-b015] Shivdasani RA, Mayer EL, Orkin SH (1995). Absence of blood formation in mice lacking the T-cell leukaemia oncoprotein tal-1/SCL. Nature.

[pgen-0030140-b016] Robb L, Lyons I, Li R, Hartley L, Kontgen F (1995). Absence of yolk sac hematopoiesis from mice with a targeted disruption of the scl gene. Proc Natl Acad Sci U S A.

[pgen-0030140-b017] Visvader JE, Fujiwara Y, Orkin SH (1998). Unsuspected role for the T-cell leukemia protein SCL/tal-1 in vascular development. Genes Dev.

[pgen-0030140-b018] Patterson LJ, Gering M, Patient R (2005). Scl is required for dorsal aorta as well as blood formation in zebrafish embryos. Blood.

[pgen-0030140-b019] Dooley KA, Davidson AJ, Zon LI (2005). Zebrafish scl functions independently in hematopoietic and endothelial development. Dev Biol.

[pgen-0030140-b020] Juarez MA, Su F, Chun S, Kiel MJ, Lyons SE (2005). Distinct roles for SCL in erythroid specification and maturation in zebrafish. J Biol Chem.

[pgen-0030140-b021] Habeck H, Odenthal J, Walderich B, Maischein H, Schulte-Merker S (2002). Analysis of a zebrafish VEGF receptor mutant reveals specific disruption of angiogenesis. Curr Biol.

[pgen-0030140-b022] Lawson ND, Weinstein BM (2002). In vivo imaging of embryonic vascular development using transgenic zebrafish. Dev Biol.

[pgen-0030140-b023] Jin SW, Beis D, Mitchell T, Chen JN, Stainier DY (2005). Cellular and molecular analyses of vascular tube and lumen formation in zebrafish. Development.

[pgen-0030140-b024] Kumar S, Tamura K, Nei M (2004). MEGA3: Integrated software for molecular evolutionary genetics analysis and sequence alignment. Brief Bioinform.

[pgen-0030140-b025] Prince VE, Moens CB, Kimmel CB, Ho RK (1998). Zebrafish hox genes: Expression in the hindbrain region of wild-type and mutants of the segmentation gene, valentino. Development.

[pgen-0030140-b026] Liao EC, Paw BH, Oates AC, Pratt SJ, Postlethwait JH (1998). SCL/Tal-1 transcription factor acts downstream of cloche to specify hematopoietic and vascular progenitors in zebrafish. Genes Dev.

[pgen-0030140-b027] Detrich HW, Kieran MW, Chan FY, Barone LM, Yee K (1995). Intraembryonic hematopoietic cell migration during vertebrate development. Proc Natl Acad Sci U S A.

[pgen-0030140-b028] Lieschke GJ, Oates AC, Paw BH, Thompson MA, Hall NE (2002). Zebrafish SPI-1 (PU.1) marks a site of myeloid development independent of primitive erythropoiesis: implications for axial patterning. Dev Biol.

[pgen-0030140-b029] Kalev-Zylinska ML, Horsfield JA, Flores MV, Postlethwait JH, Vitas MR (2002). Runx1 is required for zebrafish blood and vessel development and expression of a human RUNX1-CBF2T1 transgene advances a model for studies of leukemogenesis. Development.

[pgen-0030140-b030] Zhong TP, Rosenberg M, Mohideen MA, Weinstein B, Fishman MC (2000). gridlock, an HLH gene required for assembly of the aorta in zebrafish. Science.

[pgen-0030140-b031] Liao W, Bisgrove BW, Sawyer H, Hug B, Bell B (1997). The zebrafish gene cloche acts upstream of a flk-1 homologue to regulate endothelial cell differentiation. Development.

[pgen-0030140-b032] Lawson ND, Scheer N, Pham VN, Kim CH, Chitnis AB (2001). Notch signaling is required for arterial-venous differentiation during embryonic vascular development. Development.

[pgen-0030140-b033] Song HD, Sun XJ, Deng M, Zhang GW, Zhou Y (2004). Hematopoietic gene expression profile in zebrafish kidney marrow. Proc Natl Acad Sci U S A.

[pgen-0030140-b034] Berdougo E, Coleman H, Lee DH, Stainier DY, Yelon D (2003). Mutation of weak atrium/atrial myosin heavy chain disrupts atrial function and influences ventricular morphogenesis in zebrafish. Development.

[pgen-0030140-b035] Dong PD, Munson CA, Norton W, Crosnier C, Pan X (2007). Fgf10 regulates hepatopancreatic ductal system patterning and differentiation. Nat Genet.

[pgen-0030140-b036] Lejeune F, Maquat LE (2005). Mechanistic links between nonsense-mediated mRNA decay and pre-mRNA splicing in mammalian cells. Curr Opin Cell Biol.

[pgen-0030140-b037] Mably JD, Mohideen MA, Burns CG, Chen JN, Fishman MC (2003). heart of glass regulates the concentric growth of the heart in zebrafish. Curr Biol.

[pgen-0030140-b038] Chang CP, Neilson JR, Bayle JH, Gestwicki JE, Kuo A (2004). A field of myocardial-endocardial NFAT signaling underlies heart valve morphogenesis. Cell.

[pgen-0030140-b039] Ema M, Takahashi S, Rossant J (2006). Deletion of the selection cassette, but not cis-acting elements, in targeted Flk1-lacZ allele reveals Flk1 expression in multipotent mesodermal progenitors. Blood.

[pgen-0030140-b040] Motoike T, Markham DW, Rossant J, Sato TN (2003). Evidence for novel fate of Flk1+ progenitor: contribution to muscle lineage. Genesis.

[pgen-0030140-b041] Rottbauer W, Just S, Wessels G, Trano N, Most P (2005). VEGF-PLCgamma1 pathway controls cardiac contractility in the embryonic heart. Genes Dev.

[pgen-0030140-b042] Thompson MA, Ransom DG, Pratt SJ, MacLennan H, Kieran MW (1998). The cloche and spadetail genes differentially affect hematopoiesis and vasculogenesis. Dev Biol.

[pgen-0030140-b043] Covassin LD, Villefranc JA, Kacergis MC, Weinstein BM, Lawson ND (2006). Distinct genetic interactions between multiple Vegf receptors are required for development of different blood vessel types in zebrafish. Proc Natl Acad Sci U S A.

[pgen-0030140-b044] Trinh LA, Stainier DY (2004). Fibronectin regulates epithelial organization during myocardial migration in zebrafish. Dev Cell.

[pgen-0030140-b045] Patterson LJ, Gering M, Eckfeldt CE, Green AR, Verfaillie CM (2006). The transcription factors, Scl and Lmo2, act together during development of the haemangioblast in zebrafish. Blood.

[pgen-0030140-b046] Dehal P, Boore JL (2005). Two rounds of whole genome duplication in the ancestral vertebrate. PLoS Biol.

[pgen-0030140-b047] Ferrier DE, Dewar K, Cook A, Chang JL, Hill-Force A (2005). The chordate ParaHox cluster. Curr Biol.

[pgen-0030140-b048] Duret L, Chureau C, Samain S, Weissenbach J, Avner P (2006). The Xist RNA gene evolved in eutherians by pseudogenization of a protein-coding gene. Science.

[pgen-0030140-b049] Cooper DW (1993). X-inactivation in marsupials and monotremes. Seminars in Developmental Biology.

[pgen-0030140-b050] van Nes J, de Graaff W, Lebrin F, Gerhard M, Beck F (2006). The Cdx4 mutation affects axial development and reveals an essential role of Cdx genes in the ontogenesis of the placental labyrinth in mice. Development.

[pgen-0030140-b051] Ema M, Faloon P, Zhang WJ, Hirashima M, Reid T (2003). Combinatorial effects of Flk1 and Tal1 on vascular and hematopoietic development in the mouse. Genes Dev.

[pgen-0030140-b052] Sugi Y, Markwald RR (1996). Formation and early morphogenesis of endocardial endothelial precursor cells and the role of endoderm. Dev Biol.

[pgen-0030140-b053] Serbedzija GN, Chen JN, Fishman MC (1998). Regulation in the heart field of zebrafish. Development.

[pgen-0030140-b054] Hsieh PC, Davis ME, Lisowski LK, Lee RT (2006). Endothelial-cardiomyocyte interactions in cardiac development and repair. Annu Rev Physiol.

